# MSCs-derived apoptotic extracellular vesicles promote muscle regeneration by inducing Pannexin 1 channel-dependent creatine release by myoblasts

**DOI:** 10.1038/s41368-022-00205-0

**Published:** 2023-01-16

**Authors:** Qingyuan Ye, Xinyu Qiu, Jinjin Wang, Boya Xu, Yuting Su, Chenxi Zheng, Linyuan Gui, Lu Yu, Huijuan Kuang, Huan Liu, Xiaoning He, Zhiwei Ma, Qintao Wang, Yan Jin

**Affiliations:** 1 State Key Laboratory of Military Stomatology & National Clinical Research Center for Oral Diseases& Shaanxi International Joint Research Center for Oral Diseases, Center for Tissue Engineering, School of Stomatology, Air Force Medical University, Xi’an, China; 2 State Key Laboratory of Military Stomatology & National Clinical Research Center for Oral Diseases & Shaanxi Key Laboratory of Stomatology, Digital Dentistry Center, School of Stomatology, Air Force Medical University, Xi’an, China; 3 State Key Laboratory of Military Stomatology & National Clinical Research Center for Oral Diseases & Shaanxi Clinical Research Center for Oral Diseases, Department of Preventive Dentistry, School of Stomatology, Air Force Medical University, Xi’an, China; 4 State Key Laboratory of Military Stomatology& National Clinical Research Center for Oral Diseases & Shaanxi Engineering Research Center for Dental Materials and Advanced Manufacture, Department of Periodontology, School of Stomatology, Air Force Medical University, Xi’an, China; 5 State Key Laboratory of Military Stomatology & National Clinical Research Center for Oral Diseases & Shaanxi Key Laboratory of Oral Diseases, Department of Prosthodontics, School of Stomatology, Air Force Medical University, Xi’an, China; 6 Aerospace Clinical Medical Center, School of Aerospace Medicine, Air Force Medical University, Xi’an, China; 7 Department of Otolaryngology Head and Neck Surgery, Peking University Third Hospital, Beijing, China

**Keywords:** Apoptosis, Regeneration, Molecular medicine

## Abstract

Severe muscle injury is hard to heal and always results in a poor prognosis. Recent studies found that extracellular vesicle-based therapy has promising prospects for regeneration medicine, however, whether extracellular vesicles have therapeutic effects on severe muscle injury is still unknown. Herein, we extracted apoptotic extracellular vesicles derived from mesenchymal stem cells (MSCs-ApoEVs) to treat cardiotoxin induced tibialis anterior (TA) injury and found that MSCs-ApoEVs promoted muscles regeneration and increased the proportion of multinucleated cells. Besides that, we also found that apoptosis was synchronized during myoblasts fusion and MSCs-ApoEVs promoted the apoptosis ratio as well as the fusion index of myoblasts. Furthermore, we revealed that MSCs-ApoEVs increased the relative level of creatine during myoblasts fusion, which was released via activated Pannexin 1 channel. Moreover, we also found that activated Pannexin 1 channel was highly expressed on the membrane of myoblasts-derived ApoEVs (Myo-ApoEVs) instead of apoptotic myoblasts, and creatine was the pivotal metabolite involved in myoblasts fusion. Collectively, our findings firstly revealed that MSCs-ApoEVs can promote muscle regeneration and elucidated that the new function of ApoEVs as passing inter-cell messages through releasing metabolites from activated Pannexin 1 channel, which will provide new evidence for extracellular vesicles-based therapy as well as improving the understanding of new functions of extracellular vesicles.

## Introduction

Skeletal muscles are one pivotal organ in human body, accounting for 35%–45% of body weights, with indispensable roles in the motion system^1,2^. Muscle injuries occur frequently in daily life and mild muscle trauma could be cured without resulting in a poor prognosis, however, severe muscle trauma always led to muscle degeneration and dysfunction^3^. During muscle regeneration process after trauma, the improper regeneration of myofibers often caused decreased functional capacity of muscle, resulting in muscle atrophy or necrosis, diminishing functional capacity, and compromising life quality in consequence^4–6^.

Muscle flaps were widely used for severe muscle injured for a long time, however, the limited sources of muscle flaps as well as the secondary trauma caused by obtaining muscle flaps cannot be ignored^7^. Recently, mesenchymal stem cells (MSCs)-based therapy has shown promising prospects for severe muscle trauma. Several studies used MSCs transplantation to treat injured muscles and found that MSCs transplantation could greatly improve the regeneration capacity of muscle tissue^8–10^. However, only part of the implanted MSCs could differentiate into muscle cells or regulate functions of adjacent cells via paracrine, the majority of exogenous MSCs will undergo apoptosis and then exert multi roles through interacting with adjacent cells when they are transplanted into host^11–14^. Apoptotic extracellular vesicles (ApoEVs) are usually generated during apoptosis via blebbing or budding^15,16^, and our previous studies had demonstrated that ApoEVs had intriguing features as therapeutic agents, such as ameliorating osteopenia, improving myocardial infarction, promoting cutaneous wound healing, and ameliorating type 2 diabetes^17–20^. However, whether MSCs-ApoEVs have therapeutic effects on severe muscle injury remained unknown.

Thus, in our present study, we used MSC-derived ApoEVs (MSCs-ApoEVs) to treat muscle injury and further investigated the underlying mechanisms behind it. We found that MSCs-ApoEVs promote muscle regeneration both in vivo and in vitro, and we found that MSCs-ApoEVs promote myoblasts releasing Myo-ApoEVs by increasing the apoptosis ratio of fusing myoblasts, which enables metabolite creatine release from the activated Pannexin 1 channel and promote myoblasts fusion in consequence. Our findings firstly elucidated the new function of ApoEVs as passing inter-cell messages through releasing metabolite from activated Pannexin 1 channel, which will shed light on extracellular vesicle-based therapy and the understanding of new functions of extracellular vesicles.

## Results

### Characterization of ApoEVs from bone marrow-derived MSCs (MSCs-ApoEVs)

**Fig. 1:**
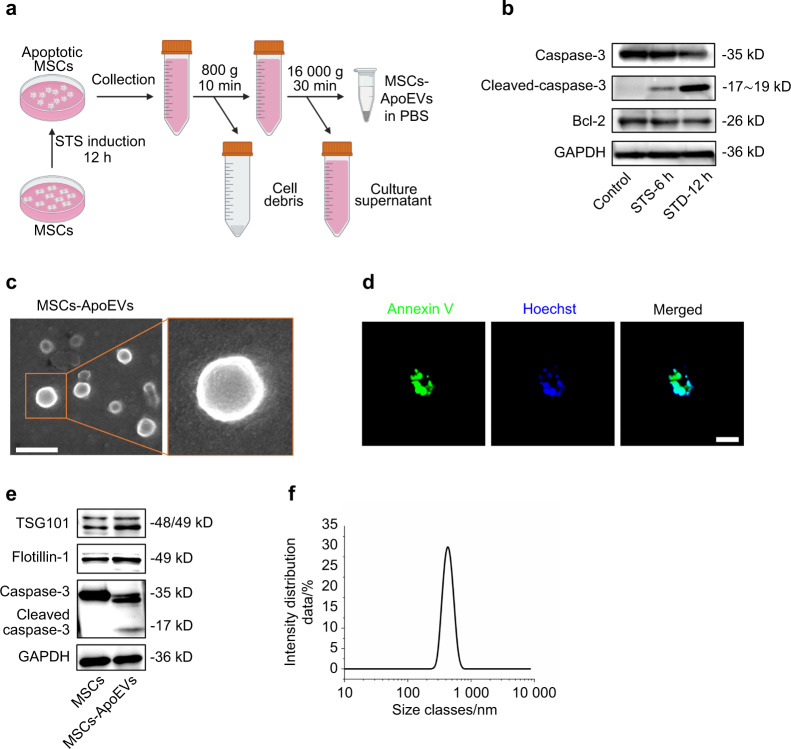
Characterization of MSC-derived ApoEVs. **a** Flow diagram of extracting MSC-derived ApoEVs. **b** The expression of apoptosis-related protein Caspase-3, Cleaved-Caspase-3, and BCL-2 of apoptotic MSCs examined by western blot. **c** The representative images of MSCs-ApoEVs examined by SEM, wide-field, and local magnification, the scale bar indicates 500 nm. **d** The representative fluorescence images for FITC-conjugated Annexin V staining for phosphatidylserine and Hoechst staining for DNA fragments of MSCs-ApoEVs, the scale bar indicates 10 μm. **e** The expression of EVs-enriched protein TSG101 and Flotillin-1 with apoptosis-related proteins caspase-3 and cleaved caspase-3 of MSCs and MSCs-ApoEVs examined by western blot. **f** The size distribution of MSC-ApoEVs was examined by DLS. STS staurosporine, DLS dynamic light scattering, SEM scanning electron microscope

MSCs isolated from 8-week female C57BL/6 J mice positively expressed the MSCs surface markers CD105, CD90, and CD73, while negatively expressed the hematopoietic stem cells surface marker CD 45 (Supplementary Fig. 1a). Besides that, the isolated MSCs have osteogenic and adipogenic differentiation potentials as well as clone-forming ability (Supplementary Fig. 1b–d). Next, we induced MSCs undergoing apoptosis by 0.5 μmol·L^−1^ Staurosporine (STS) and separated MSC-derived ApoEVs by differential centrifugation (Fig. 1a) and western blot results indicated the apoptosis-related protein such as Caspase-3 and Bcl-2 were activated after STS was applied (Fig. 1b). The isolated MSCs-ApoEVs are spherically shaped under SEM observation (Fig. 1c) and expressed the apoptosis-specific marker phosphatidylserine on their surface with DNA fragments in their contents (Fig. 1d). Moreover, the isolated MSCs-ApoEVs highly expressed EVs-enriched proteins such as TSG101 and Flotillin-1 with apoptosis-specific protein cleaved caspase-3 (Fig. 1e). In addition, diameters of MSCs-ApoEVs are among 100–1 000 nm by DLS analysis (Fig. 1f).

### MSCs-ApoEVs promote muscle regeneration of the tibialis anterior (TA) injury model

**Fig. 2:**
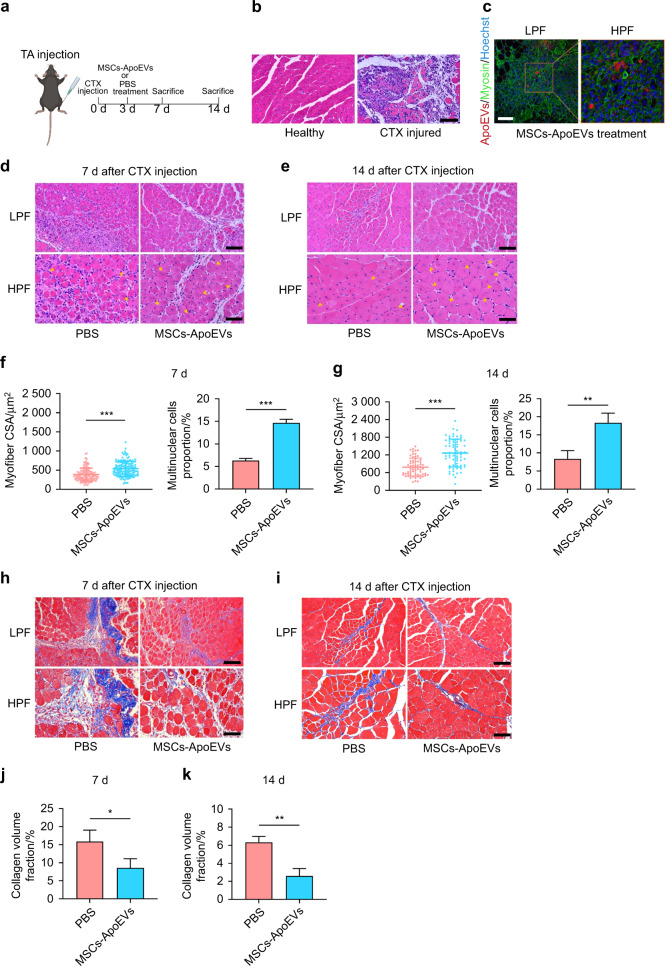
MSCs-ApoEVs promote muscle regeneration of TA injury. **a** The schematic graph of establishment and treatment for TA injury model. **b** The representative images of the TA sample by H&E staining after 3 days’ CTX injection. **c** The representative fluorescence images of the TA sample after MSCs-ApoEVs treatment, MSCs-ApoEVs were pre-stained by PKH26, the scale bar indicates 100 μm. **d**, **e** The representative images of the TA sample by H&E staining after PBS or MSCs-ApoEVs treatment on Day 7 or Day 14, yellow arrows indicated multinucleated cells. **f**, **g** Analysis of myofiber CSA and multinuclear cells proportion of different treated groups in Day 7 or Day 14 **h**, **i** The representative images and analysis of the TA sample by Masson staining after PBS or MSCs-ApoEVs treatment on Day 7 or Day 14. **j**, **k** Analysis of collagen volume fraction of different treated groups on Day 7 or Day 14. *n* = 3 per group; the scale bar indicated 100 μm in the low magnification field and 50 μm in the high magnification field. Data were shown as mean ± SD; ns not significant; *P < 0.05, **P < 0.01, ***P < 0.001. TA tibialis anterior, CTX cardiotoxin, H&E hematoxylin & eosin, LPF low power field, HPF high power field, CSA cross-sectional area

We established mice acute TA injury model by in situ cardiotoxin (CTX) injection and treated with MSCs-ApoEVs subsequently (Fig. 2a). The H&E staining of TA tissue 3 d after CTX injection showed that normal myofibers were damaged and a large number of inflammatory cells were infiltered (Fig. 2b). Then, we examined whether MSCs-ApoEVs could be phagocyted by myoblasts in vivo and found that PKH26 labeled MSCs-ApoEVs (red) are around myosin positive cells (green) while some labeled MSCs-ApoEVs are co-localized with myosin positive cells (Fig. 2c). After MSCs-ApoEVs treatment, the H&E staining of TA tissue 7 d and 14 d after CTX injection both showed the myofiber CSA (cross-sectional area) and the proportion of the multinuclear cells are increased compared to the PBS group (Fig. 2d–g). Moreover, MSCs-ApoEVs treatment also significantly decreased the collagen volume fraction of TA tissue in 7 d and 14 d after CTX injection compared to the control group (Fig. 2h–k).

### MSCs-ApoEVs promote fusion and apoptosis ratio of myoblasts

**Fig. 3:**
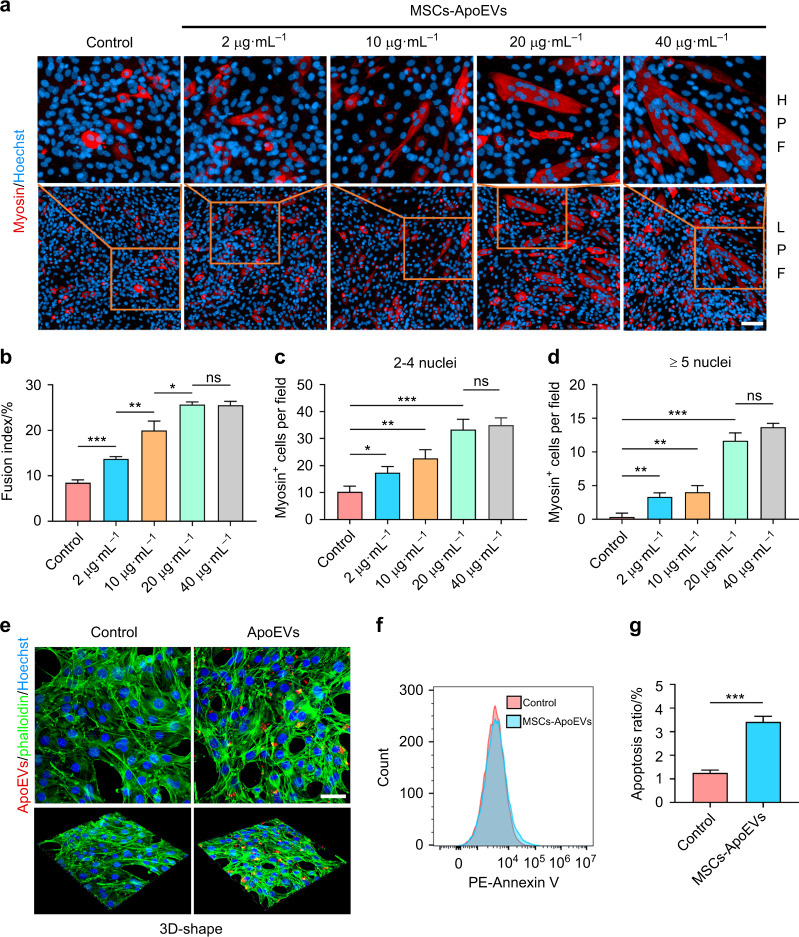
MSCs-ApoEVs promote fusion and apoptosis ratio of C2C12 myoblasts in vitro. **a**–**d** MSCs-ApoEVs promote C2C12 myoblasts fusion ratio. The representative immunofluorescence images of myosin staining of C2C12 myoblasts in control or MSCs-ApoEVs treated groups, scale bar indicates 20 μm (**a**). The analysis of fusion index and the number of myosin^+^ multinucleated cells per field in different treated groups (**b**–**d**). **e** The representative fluorescence images of C2C12 myoblasts after MSCs-ApoEVs treatment, MSCs-ApoEVs were pre-stained by PKH26, scale bar indicates 50 μm. **f**, **g** MSCs-ApoEVs promote apoptosis ratio of C2C12 myoblasts in the fusion medium. The relative Annexin V expression in C2C12 myoblasts of control group or MSCs-ApoEVs treated group (**f**). The analysis of apoptosis ratio of C2C12 myoblasts in different treated groups (**g**). *n* = 3 per group; data were shown as mean ± SD; ns, not significant; *P < 0.05, **P < 0.01, ***P < 0.001. LPF low power field, HPF high power field

After we observed the therapeutic effects of MSCs-ApoEVs on CTX-induced TA injury in vivo, we wondered how MSCs-ApoEVs exert their therapeutic effects. We used MSCs-ApoEVs to treat C2C12 myoblasts and examined the fusion index after application. The results of immunofluorescence staining indicated that the fusion index and the number of multinucleated cells of C2C12 myoblasts were significantly increased after MSCs-ApoEVs were applied and strongly related to the concentration of MSCs-ApoEVs **(**Fig. 3a–d**)**. Next, we examined whether ApoEVs could be phagocyted by C2C12 myoblasts in vitro and found that PKH26 labeled ApoEVs (red) are co-localized with C2C12 myoblasts after 3 h treatment **(**Fig. 3e**)**. Since the previous study denoted that apoptosis played an important role in the fusion process of C2C12 myoblasts, we further investigated the apoptosis ratio of C2C12 myoblasts when treated with MSCs-ApoEVs by flow cytometry and found that the apoptosis ratio of C2C12 myoblasts in fusion medium is significantly raised compared to the control group **(**Fig. 3f–g**)**.

### Myoblast-derived apoptotic extracellular vesicles (Myo-ApoEVs) are generated during myoblasts fusion

**Fig. 4:**
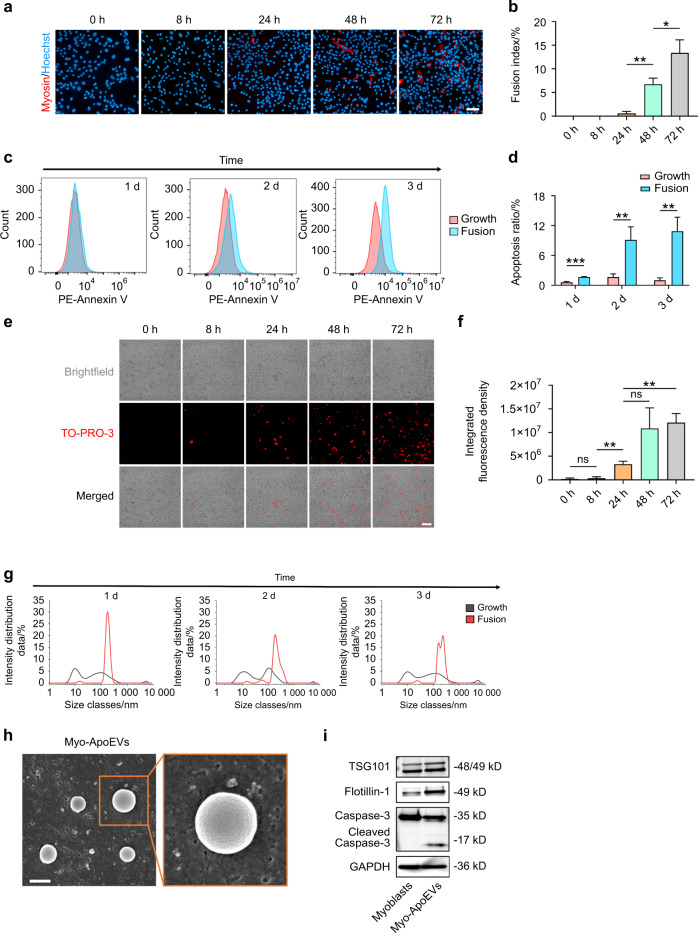
Myo-ApoEVs are generated during C2C12 myoblasts fusion process. **a**, **b** The fusion of C2C12 myoblasts increased over time. The immunofluorescence images of myosin staining of C2C12 myoblasts in fusion medium over time (**a**). The analysis of fusion index of C2C12 myoblasts in fusion medium over time (**b**). **c**, **d** The Apoptosis ratio of C2C12 myoblasts in the fusion medium was increased over time. The relative Annexin V expression of C2C12 myoblasts in fusion medium over time (**c**). The analysis of apoptosis ratio of C2C12 myoblasts in fusion medium over time (**d**). **e**, **f** Myo-ApoEVs are generated during C2C12 myoblasts fusion process over time. The representative immunofluorescence images of TO-PRO-3 staining of Myo-ApoEVs in fusion medium over time (**e**). The analysis of integrated fluorescence density of Myo-ApoEVs in fusion medium over time (**f**). **g** The size distribution of vesicles in supernatants of growth or fusion medium was examined by DLS. **h** The representative image of Myo-ApoEVs examined by SEM, wide-field and local magnification. **i** The expression of EVs-enriched protein TSG101 and Flotillin-1 with apoptosis-related proteins caspase-3 and cleaved caspase-3 of C2C12 myoblasts and Myo-ApoEVs examined by western blot. *n* = 3 per group; data were shown as mean ± SD; ns, not significant; the scale bar indicates 20 μm in the image **a**, **e**, and 500 nm in the image **h**; *P < 0.05, **P < 0.01, ***P < 0.001. DLS dynamic light scattering, SEM scanning electron microscope

To further investigate the underlying mechanisms of the fusion process of C2C12 myoblasts, we observed the dynamic changes of C2C12 myoblasts in the fusion medium and found that the fusion index of C2C12 myoblasts increased over time (Fig. 4a, b). Meanwhile, we examined the apoptosis ratio of C2C12 myoblasts in the fusion process and found that the apoptosis ratio indicated by Annexin V was significantly increased over time (Fig. 4c, d). Then, we observed the fusion process of C2C12 myoblasts by time-lapse microscopy from 0 h to 72 h and surprisingly found that the number of TO-PRO-3 labeled Myo-ApoEVs were raised over time (Fig. 4e, f, Video 1). Moreover, we also examined the supernatants of C2C12 myoblasts in growth or fusion medium by Dynamic Light Scattering (DLS) and found that compare to the growth medium group, there was a major peak at 100–1000 nm in DLS results of the fusion medium in Day 1–Day 3, suggesting the existence of Myo-ApoEVs are generated during C2C12 myoblasts fusion process (Fig. 4g). Finally, we used differential centrifugation to extract Myo-ApoEVs and used SEM to observe the morphology of Myo-ApoEVs subsequently. The SEM results showed that Myo-ApoEVs were spherical, and their diameters were about 500 nm (Fig. 4h). In addition, we also examined whether Myo-ApoEVs also expressed EVs-enriched proteins and apoptosis-specific proteins and found that Myo-ApoEVs highly expressed TSG101 and Flotillin-1 with cleaved caspase-3 (Fig. 4i).

### Inhibition of apoptosis in myoblasts impaired the fusion process

**Fig. 5:**
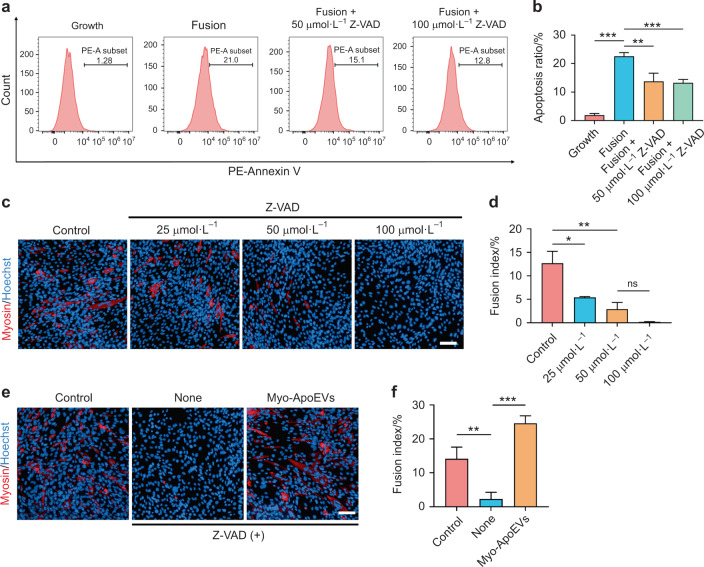
Inhibition of apoptosis in C2C12 myoblasts impaired the fusion process. **a**, **b** Z-VAD inhibited the apoptosis ratio of C2C12 myoblasts in the fusion medium. The relative PE-Annexin V expression of C2C12 myoblasts in the growth medium, fusion medium, and fusion medium with Z-VAD was examined by flow cytometry (**a**). The analysis of apoptosis ratio of C2C12 myoblasts in different treated groups (**b**). **c**, **d** Z-AVD impaired the fusion ratio of C2C12 myoblasts in the fusion medium. The representative immunofluorescence images of myosin staining of C2C12 myoblasts in control and Z-VAD treated groups (**c**). The analysis of the fusion index of C2C12 myoblasts in different treated groups (**d**). **e**, **f** Myo-ApoEVs rescue Z-VAD impaired fusion process of C2C12 myoblasts. The representative immunofluorescence images of myosin staining of C2C12 myoblasts in control and Z-VAD treated groups with or without Myo-ApoEVs (**e**). The analysis of fusion index of C2C12 myoblasts in different treated groups (**f**). *n* = 3 per group; data were shown as mean ± SD; ns not significant; the scale bar indicates 20 μm; *P < 0.05, **P < 0.01, ***P < 0.001

To further unveil the roles apoptosis played in the process of C2C12 myoblasts fusion, we investigated the fusion index of C2C12 myoblasts when apoptosis was inhibited. We used Z-VAD to inhibit the apoptosis of C2C12 myoblasts and found that apoptosis ratio of C2C12 myoblasts indicated by PE-conjugated Annexin V in fusion medium at Day 3 was significantly decreased (Fig. 5a, b). Then, the fusion index of C2C12 myoblasts was significantly decreased when Z-VAD was applied, indicating the fusion of C2C12 myoblasts is highly synchronized with the apoptosis of C2C12 myoblasts in the fusion process. In addition, the fusion index of C2C12 myoblasts was also inhibited by using sertraline (Supplementary Fig. 2), an antidepressant which reported to block the formation of apoptotic vesicles^21^. Moreover, we used Myo-ApoEVs to rescue the impaired fusion process of C2C12 myoblasts, whose apoptosis is inhibited by Z-VAD (100 μmol·L^−1^) and found that the fusion index of C2C12 myoblasts was significantly increased (Fig. 5e, f), which in turn suggesting Myo-ApoEVs plays indispensable roles in myoblasts fusion.

### Creatine was released from activated Pannexin 1 channel on Myo-ApoEVs

**Fig. 6:**
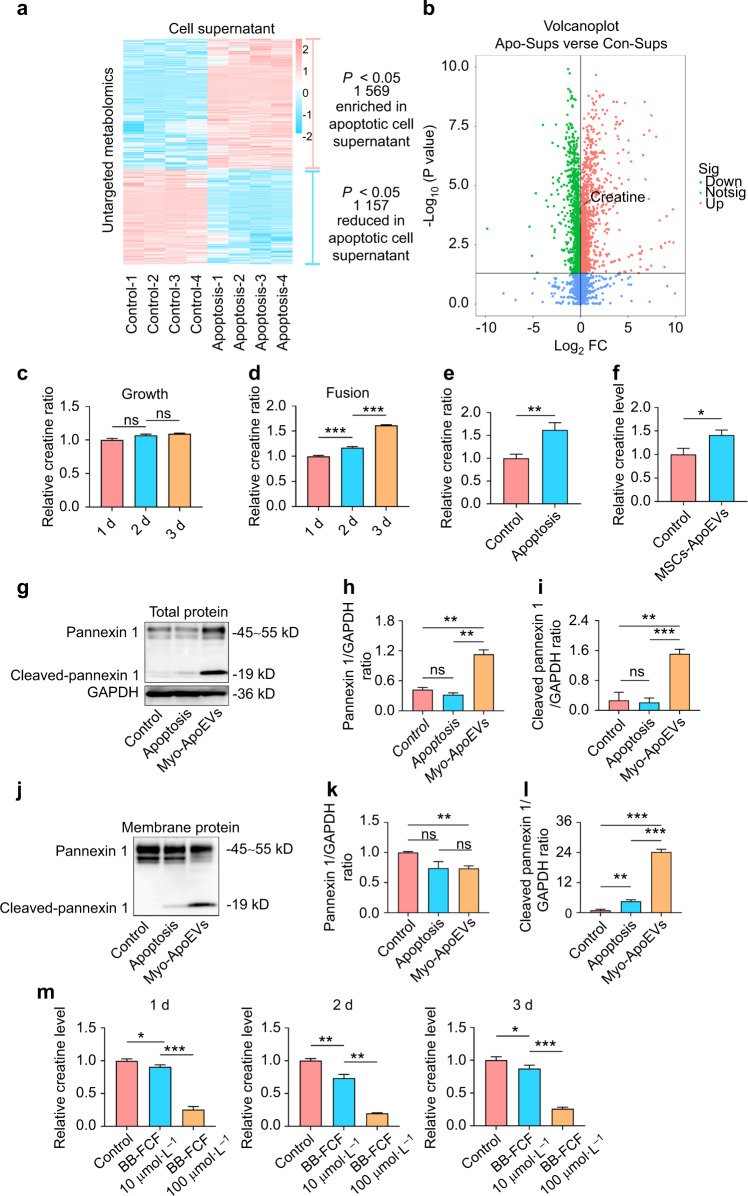
Creatine is released from activated Pannexin 1 channel of Myo-ApoEVs during C2C12 myoblasts fusion. **a**, **b** Heat map (**a**) and volcano plot (**b**) of untargeted metabolomics of supernatants from control group or STS-induced apoptotic C2C12 myoblasts group, representing statistically enriched or reduced (*P* < 0.05) metabolites in the apoptotic supernatant relative to the control supernatant, *n* = 4 per group. **c**–**f** Relative creatine ratio in different treated C2C12 myoblasts groups. Relative creatine change of C2C12 myoblasts supernatants in growth medium (**c**) or fusion medium (**d**) over time. Relative creatine change of C2C12 myoblasts supernatants in the STS-induced apoptotic group relative to the control group (**e**). Relative creatine change of C2C12 myoblasts supernatants in the MSCs-ApoEVs treated group relative to the control group (**f**). **g**–**l** Relative expression and analysis of Pannexin 1 channel protein of control group, 12 h STS-induced group, and Myo-ApoEVs group in whole cell protein lysis or membrane protein lysis. Relative expression of Pannexin 1 channel of different groups in whole cell protein lysis or membrane protein lysis examined by western blot (**g**, **j**). Analysis of relative expression of Pannexin 1 protein and cleaved-Pannexin 1 protein of different groups in whole cell protein lysis or membrane protein lysis (**h**, **i**, **k**, **l**). **m** Relative creatine change of C2C12 myoblasts supernatants in fusion medium over time with BB-FCF treatment. *n* = 3 per group; data were shown as mean ± SD; ns, not significant; *P < 0.05; **P < 0.01; ***P < 0.001. BB-FCF, Brilliant Blue FCF

To understand the roles of Myo-ApoEVs in C2C12 myoblasts fusion process, we examined the supernatants derived from normal or apoptotic C2C12 myoblasts using untargeted metabolomics. The untargeted metabolomics results showed that compared to the control group, 1569 kinds of metabolites were increased, and 1157 kinds of metabolites were decreased in the supernatants from apoptotic C2C12 myoblasts (Fig. 6a). Among those metabolites, creatine, a compound derived from amino acids which could promote C2C12 myoblasts fusion^22,23^, was significantly increased in supernatants of apoptotic C2C12 myoblasts (Fig. 6b). Then, we examined the change of relative expression of creatine in supernatants of C2C12 myoblasts in growth or fusion process and found that the relative expression of creatine was significantly raised in fusion medium over time while that was barely changed in growth medium (Fig. 6c, d). Besides that, we examined the relative expression of creatine in supernatants of STS-induced apoptotic C2C12 myoblasts and MSCs-ApoEVs treated C2C12 myoblasts and found that the relative expressions of creatine were both significantly increased (Fig. 6e, f). Pannexin 1 is an oligomeric membrane channel, which could allow metabolites such as ATP, creatine, glutamate, and spermidine released when its C terminus is cleaved by caspase 3 or 7 during apoptosis^24–26^. We examined the expression of Pannexin 1 channel in whole protein lysis and found that when compared to C2C12 myoblasts or STS-induced apoptotic C2C12 myoblasts, the expression of total Pannexin 1 and cleaved Pannexin 1 is greatly increased in Myo-ApoEVs (Fig. 6g–i). Furthermore, we examined the relative expression of Pannexin 1 channel in membrane protein lysis, and the results showed that although total Pannexin 1 protein was expressed among three groups at similar levels, cleaved Pannexin 1 protein was mainly expressed on the membrane protein of Myo-ApoEVs (Fig. 6j–l), which indicating creatine was released to extracellular microenvironment through activated Pannexin 1 channel of Myo-ApoEVs in the fusion process. Moreover, we blocked the functions of Pannexin 1 channel by using the BB-FCF, a selective inhibitor of Pannexin 1 channels^27,28^, and found that the relative expression of creatine was significantly decreased in the supernatant of C2C12 myoblasts in fusion medium at Day 1, Day 2 and Day 3 (Fig. 6m), also suggesting creatine was released via activated Pannexin 1 channel in C2C12 myoblasts fusion process.

### Creatine is necessary for promoting the myoblasts fusion process

**Fig. 7:**
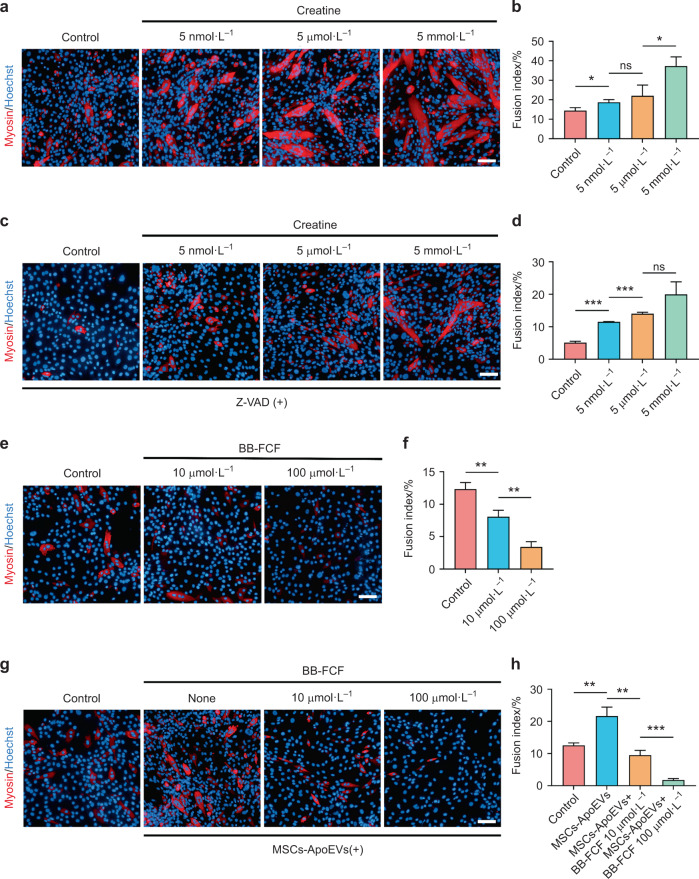
Creatine promotes C2C12 myoblasts fusion in vitro. **a**–**d** The representative immunofluorescence images of myosin staining of C2C12 myoblasts in control and creatine-treated groups with or without Z-VAD exist (**a**, **c**). The analysis of fusion index of C2C12 myoblasts in different treated groups (**b**, **d**). **e**, **f** The representative immunofluorescence images and analysis of fusion index of C2C12 myoblasts in control and Pannexin 1 inhibitor BB-FCF treated groups. **g**, **h** The representative immunofluorescence images and analysis of fusion index of C2C12 myoblasts in control and BB-FCF and MSCs-ApoEVs treated groups; *n* = 3 per group; data were shown as mean ± SD; ns not significant, the scale bar indicates 20 μm; *P < 0.05, **P < 0.01, ***P < 0.001

To further explore the functions of creatine in C2C12 myoblasts fusion, we added exogenous creatine during C2C12 myoblasts fusion process and found that the fusion index of C2C12 myoblasts was significantly raised with the concentration of creatine increasing (Fig. 7a, b). Next, we examined whether exogenous creatine could rescue the impaired fusion process of C2C12 myoblasts, which is inhibited by Z-VAD, and found that the fusion index of C2C12 myoblasts was rescued and raised with the concentration of exogenous creatine increasing (Fig. 7c, d). Furthermore, we blocked the functions of Pannexin 1 channel by using the BB-FCF and found that the fusion index of C2C12 myoblasts was significantly inhibited (Fig. 7e, f). In addition, we also examined whether MSCs-ApoEVs could rescue the fusion of C2C12 myoblasts when the Pannexin 1 channel was inhibited. The results showed that MSCs-ApoEVs failed to rescue the fusion of C2C12 myoblasts when BB-FCF was applied (Fig. 7g, h), which indicates that MSCs-ApoEVs exert their functions only when Pannexin 1 channel is working.

**Fig. 8:**
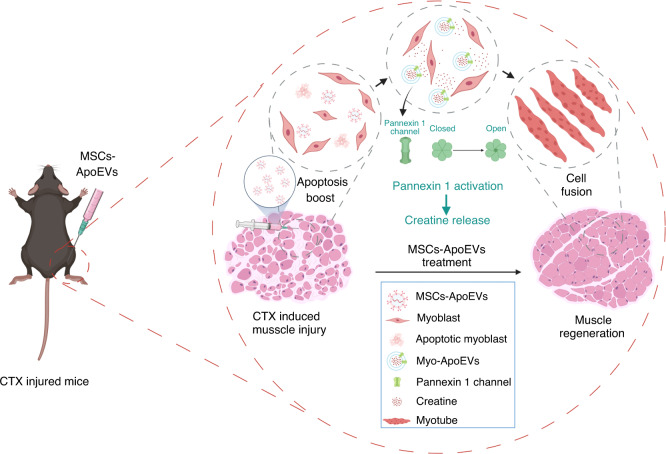
Schema for MSCs-ApoEVs promotes muscle regeneration by boosting apoptosis of myoblasts and increasing release of creatine from activated Pannexin 1 Channel on Myo-ApoEVs

To sum up, we found that MSCs-ApoEVs promote TA injury regeneration in vivo and myoblasts fusion in vitro. Mechanistically, we demonstrated the MSCs-ApoEVs boost the apoptosis of myoblasts, subsequently promoting creatine release from activated Pannexin 1 channel on Myo-ApoEVs and finally increasing the fusion of myoblasts to promote muscle regeneration (Fig. 8).

## Discussion

Apoptosis is a programmed cell death process that is delicately regulated. Previous research believed that apoptosis is a controlled biological mechanism mainly involved in early development^29,30^, however, recent studies revealed that the interaction of apoptotic cells and adjacent cells played pivotal roles in inflammation resolution, tissue remodeling, and regeneration^31–33^. In terms of myofiber formation, several studies demonstrated that apoptosis was also involved. For instance, apoptosis and the exposure of phosphatidylserine, which could be recognized by phagocytosis receptors such as BAI1 or Stabilin-2, is indispensable in myoblasts fusion to myofibers^34,35^. Since apoptosis is a dynamic process and a large amount of apoptotic extracellular vesicles (ApoEVs) are generated during this process, whether ApoEVs have effects on the myofibers formation remained unknown. Meanwhile, considering there are few myoblasts in muscle tissue when compared to differentiated muscle cells, using exogenous means to activate those myoblasts to undergo natural apoptosis and promote myoblasts subsequently fusion into mature myofibers is worth investigating. Thus, in our study, we used MSCs-ApoEVs to rescue injured muscle and found that MSCs-ApoEVs could promote the regeneration of acute TA injury in vivo as well as promote myoblasts fusion in vitro. Moreover, we found that apoptosis is highly synchronized with the fusion of myoblasts and MSCs-ApoEVs increased the apoptosis ratio of myoblasts in the initial stage of fusion, which boosts the natural process of myoblasts fusion.

Extracellular vesicles (EVs) are a large group of vesicle-like structures released by cells into the extracellular microenvironment, which are involved in cell–cell communication and many pivotal biological processes^36,37^. Current studies mainly focused on the transport function of extracellular vesicles such as carrying and transporting RNA, DNA, proteins, or lipids into host cells^38,39^, however, whether there are other interesting features of extracellular vesicles remains unknown. ApoEVs are generated through the characteristic blebbing and budding of the apoptotic cell membrane during apoptosis^40^ and exhibited therapeutic potentials such as resolving inflammation and promoting regeneration in recent years^19,20^. In our study, we found that ApoEVs generated in myoblasts fusion process exerted intriguing function, which enable creatine generated in apoptosis of myoblasts to release from activated Pannexin 1 channel on the membrane of ApoEVs and subsequently promote the fusion of myoblasts.

A recent study demonstrated that different types of cells could release a series of conserved metabolites such as spermidine, AMP, GMP, creatine, G3P, and ATP during apoptosis through activated Pannexin 1 channel, indicating that apoptotic cells would release metabolites to actively modulate other adjacent cells rather than passively waiting to be recognized and digested by phagocytes^41^. Pannexin 1 is a widely expressed homo-heptameric membrane channel that can be reversibly activated by several mechanisms such as stretch, elevated K^+^, and ionotropic and metabotropic receptor signaling^28,42,43^. During the apoptosis process, the C terminus of Pannexin 1 protein is cleaved by caspase, leading to irreversible activation of Pannexin 1 channel and metabolites release^25,44^. However, previous studies mainly focused on the Pannexin 1 channel on apoptotic cells^45,46^ and the aim and functions of irreversible activation of Pannexin 1 channel in apoptosis remain unknown. In our study, we found that activated Pannexin 1 channel was mainly expressed on the membranes of ApoEVs rather than apoptotic cells, suggesting forming ApoEVs could be an undiscovered way for cells to release metabolites such as creatine. Besides that, we also demonstrated that creatine release was strongly synchronized with apoptosis, which is indispensable for myoblasts fusion.

Previous studies mostly focused on the transport function of EVs such as carrying miRNA or LncRNA to transport into host cells to exert their biological functions^47,48^. However, the protein channels on membranes of EVs had been ignored for a long time. Our study denoted that MSCs-ApoEVs could promote regeneration of acute muscle injury and unveiled the underlying mechanisms behind it. Most importantly, it revealed a new function of EVs, which is acting as an inter-cell message deliver through activated Pannexin 1 channel, which will provide a new sight for understanding the formation and functions of EVs.

Moreover, since the complexity and heterogeneity of ApoEVs, which subtype of ApoEVs plays a predominant role in myoblasts fusion and muscle regeneration remain unknown. Besides that, the regulation of Pannexin 1 channel during cell apoptosis also needs further study. Using advanced equipment to examine ApoEVs generation and Pannexin 1 channel activation on sing-cell level or single-EV level are highly recommended in the future studies.

## Materials and methods

### Animals

C57BL/6 J mice were purchased from the Animal Center of the Air Force Medical University. All animal experiments were performed by following protocols approved by the Animal Care Committee of the Air Force Medical University (IRB-REV-2021027). 8-week-old C57BL/6 J mice were used for MSCs isolation, ApoEVs extraction, and the establishment and treatment of CTX injured TA models. All mice were maintained in a specific pathogen-free condition with 12:12-h day/night illumination cycle.

### Isolation and characterization of bone marrow-derived MSCs

MSCs were isolated from the tibia and femur of 8-week-old female C57BL/6 J mice according to Huang’s protocol^49^ and cultured in alpha minimum essential medium (α-MEM) (Gibco, USA) supplemented with 20% fetal bovine serum (Gibco, USA), 2 mmol·L^−1^ L-glutamine, 100 U·mL^−1^ penicillin, and 100 g·mL^−1^ streptomycin (all from Sigma, USA). The MSCs were cultured at 37 °C with a humidified atmosphere of 5% CO_2_, and the culture medium was changed every 3 days. The MSCs were digested and passaged by using 0.25% trypsin (Gibco, USA) when reached 80%–90% confluence.

The characterization of surface markers on MSCs based on flow cytometry and characterization of multidirectional differentiation ability of MSCs were referred to the previous procedure^19^. Phycoerythrin (PE)-conjugated anti-mouse CD105, CD90, CD73, and CD45 were all purchased from Biolegend (USA). Alizarin red staining was performed on MSCs after 28 days’ osteogenic induction while Oil Red O staining was performed on MSCs after 14 days’ adipogenic induction. Besides that, MSCs-colonies were stained by 0.2% crystal violet after 14 day’s culture at a low seeding density. Alizarin red, Oil Red O, and crystal violet were all purchased from Sigma (USA).

### Isolation and characterization of apoptotic extracellular vesicles (ApoEVs)

The procedure of isolating MSCs-ApoEVs is based on our previous work^20^. Briefly, 0.5 μmol·L^−1^ STS with completed culture medium containing EVs-depleted FBS (pre-ultracentrifugation at 100 000 g for 18 h) was used to induce P2 MSCs undergoing apoptosis. After 12 h induction, the supernatants of apoptotic MSCs were collected and prepared for centrifugation. The procedure for isolating MSCs-ApoEVs is using 800 g, 10 min to remove cell debris of apoptotic supernatants and then using 16 000 g, 30 min to obtain MSCs-ApoEVs. The apoptosis-related protein Bcl-2 (CST, 3498), Caspase-3 (CST, 9662), Cleaved-Caspase-3 (CST, 9661), and GAPDH (CST, 2118) were examined of apoptotic MSCs at different STS-induced time by western blot. Morphology identification of MSCs-ApoEVs was measured by SEM (Hitachi, Japan) after fixation, gradient dehydration, and gold spraying. Apoptosis-related phosphatidylserine on the surface of MSCs-ApoEVs was stained by FITC-conjugated Annexin V Apoptosis Detection Kit (BD Biosciences, 556547), and DNA fragments inside MSCs-ApoEVs were stained by Hoechst 33342 (Invitrogen, H21492) according to manufactures’ protocols and detected by laser scanning confocal microscope (Nikon, Japan). EV-enriched protein TSG101 (Abcam, ab125011) and Flotillin-1 (CST, 3253) with apoptosis-related protein caspase-3 (CST, 9662) were examined of MSCs and MSCs-ApoEVs by western blot. The size distribution of MSCs-ApoEVs was measured by Dynamic Light Scattering (DLS) using Zetasizer Nano ZSE (Malvern, UK) according to the manufacturer’s protocol.

### The establishment and treatment of cardiotoxin (CTX) induced TA injury

The procedure of establishment of CTX-induced TA injury was referred to the previous work^34^. Briefly, 50 μL 1 × 10^−5 ^mol·L^−1^ CTX (Merck, USA) was injected into the left and right TA muscles of C57BL/6 J mice on day 0. After 3 days, 30 μg MSCs-ApoEVs (20 μL) was injected into the left TA muscle while PBS (20 μL) was injected into the right TA muscle for control. The TA muscles were collected and fixed by 4% paraformaldehyde on day 3, day 7, and day 14 for histological analysis. For H&E and Masson staining, 4 μm sections were stained and at least three fields were captured by microscope (Nikon, Japan) for analysis which referred to the previous study^10,50^. Besides that, Image J software (NIH, USA) was used to calculate the cross-sectional area as well as the number of multinuclear cells of H&E-stained myofiber and the collagen volume area for each field.

### Myoblast fusion assay

C2C12 murine myoblasts were purchased from Fuheng Biotechnology (China) and used for myoblast fusion assay. C2C12 myoblasts were maintained in DMEM (Gibco, USA) with 10% FBS for growth and induced for fusion in DMEM supplemented with ITS (Sigma, USA) and 0.1% FBS. For C2C12 fusion assay, C2C12 myoblasts were seeded in CellCarrier Ultra 96-well plates (PerkinElmer, USA) and induced to fusion by changing medium from growth medium to fusion medium when cells reached 70%–80% confluency. Moreover, the fusion medium was replaced every 24 h for a total of 72 h unless specified otherwise.

Z-VAD-FMK (MCE, HY-16658B) was added into the fusion medium on day 1 for apoptosis inhibition, while BB-FCF (MCE, HY-D0915) was added into the fusion medium on each day for Pannexin 1 channel inhibition. C2C12 myoblasts-derived ApoEVs were isolated according to the separation procedure of MSCs-ApoEVs. MSCs-ApoEVs or Myo-ApoEVs were added to the fusion medium to treat C2C12 myoblasts on day 1 and changed to new fusion medium after 24 h. Creatine (Sigma, C3630) was used to rescue the fusion of C2C12 myoblasts by being added into the fusion medium each day.

### The analysis of myoblasts fusion

For measuring fusion, C2C12 myoblasts were washed and fixed with 4% paraformaldehyde for 20 min and then permeabilized in 0.1% Triton X-100 for 10 min. After blocking with goat serum (Boster, China), C2C12 myoblasts were incubated with mouse anti-myosin antibody (R&D, MAB4470) at 1:250 dilution overnight at 4 °C and then incubated with Alexa Fluor 594 labeled secondary antibody (Yeasen, 33212ES60) at 1:500 dilution for 1 h at room temperature and stained with Hoechst 33342 (Invitrogen, H21492) for 5 min at room temperature. The fluorescently stained C2C12 myoblasts were captured by Operetta CLS (PerkinElmer, USA) for analysis. The fusion index and the number of multinuclear myotubes were quantitated based on myosin^+^ cells per field by Image J software referred to the previous study^34^.

### Phagocytosis experiment in vivo and in vitro

For in vivo phagocytosis experiment, CTX-induced TA injury was established as mentioned above while MSCs-ApoEVs were pre-stained by PKH26 (Sigma, USA) according to the manufacturer’s protocol. The PKH26 labeled MSCs-ApoEVs were injected into left TA muscles 3 days after TA injury models were established. The TA muscles were collected and fixed, dehydrated, and embedded in optimal cutting temperature compound (Leica, Germany) 24 h after PKH26 labeled MSCs-ApoEVs injection. For immunofluorescence staining, 10 μm sections were incubated with mouse anti-myosin antibody (R&D, MAB4470) at 1:250 dilution overnight at 4 °C and then incubated with Alexa Fluor 488 labeled secondary antibody (Yeasen, 33112ES60) at 1:500 dilution for 2 h at room temperature and finally stained with Hoechst 33342 (Invitrogen, H21492) for 10 min at room temperature, all operations are carried out in dark environments. The fluorescence images were captured by laser scanning confocal microscope (Nikon, Japan).

For in vitro phagocytosis experiment, PKH26 labeled MSCs-ApoEVs were added to C2C12 myoblasts and incubated for 3 h. After that, C2C12 myoblasts were fixed, permeated, and then stained by 488-conjugated phalloidin (AAT Bioquest, 23115) for 1 h and Hoechst 33342 for 5 min at room temperature. The fluorescence images were captured by laser scanning confocal microscope (Nikon, Japan).

### Observation of ApoEVs generated during myoblasts fusion

To dynamic observe the ApoEVs generated during C2C12 myoblasts fusion, C2C12 cells were seeded in CellCarrier Ultra 96-well plates and cultured in the growth medium until cells reached 70%–80% confluency. After that, C2C12 myoblasts were changed into the fusion medium with 1 μmol·L^−1^ TO-PRO-3 (Thermo, T3605) to label ApoEVs through activated Pannexin 1 channel^25^. The time-lapse images were captured by Operetta CLS every 15 min at 37 °C and 5% CO_2_. Besides that, to measure the diameter of ApoEVs generated during C2C12 myoblasts fusion, the supernatants were collected from C2C12 myoblasts cultured in growth or fusion medium every day when changing the medium. The diameter of particles in supernatants of different groups was measured by DLS. The myoblasts-derived ApoEVs were isolated from the supernatants of C2C12 myoblasts in the fusion medium according to the protocol for obtaining MSCs-derived ApoEVs and observed by SEM (Hitachi, Japan). Moreover, EV-enriched protein TSG101 (Abcam, ab125011) and Flotillin-1 (CST, 3253) with apoptosis-related protein caspase-3 (CST, 9662) were examined of MSCs and MSCs-ApoEVs by western blot.

### Flow cytometric analysis for apoptosis

PE-Annexin V Apoptosis Detection Kit I (BD Biosciences, 559763) was used to measure the apoptosis ratio of C2C12 myoblasts in different groups according to the manufacturer’s protocol. Briefly, C2C12 myoblasts from different groups were digested, collected, washed, and finally resuspended in the annexin V-binding buffer and stained with PE-conjugated annexin V for 15 min. The apoptosis ratio was assessed by flow cytometry (Beckman Coulter, USA) based on the annexin V-stained C2C12 myoblasts.

### The detection of creatine release

Untargeted metabolomics analysis service was provided by QLBio (China). In brief, the supernatants were collected from normal and apoptotic C2C12 myoblasts and precipitated in methanol for analysis. Metabolite analysis was conducted by Rapid Separation LC (Thermo, USA) and Q Exactive (Thermo, USA). Besides that, the mass spectrometry data were processed based on METLIN Database, Human Metabolome Database, and ChemSpider Database.

Creatine Assay Kit (Abcam, ab65339) was used to detect the relative expression of creatine in the supernatants of C2C12 myoblasts according to the manufacturer’s protocol in the different groups. The supernatants of C2C12 myoblasts were collected from C2C12 myoblasts cultured in different determined groups and centrifuged at 1 000 g, 4 °C to remove cell debris. The fluorometric detection of creatine from different groups was measured by EnSight Multimode Plate Reader (PerkinElmer, USA).

### The analysis of Pannexin 1 channel activation

The activation of Pannexin 1 channel of the total protein lysis as well as the membrane protein lysis of normal C2C12 myoblasts, apoptotic C2C12 myoblasts, and Myo-ApoEVs was measured by Western Blot. The total proteins from C2C12 myoblasts were extracted by RIPA buffer (Beyotime, China) with protease inhibitor (MCE, USA) while the membrane proteins were extracted by Membrane Protein Extraction Kit (Thermo, 89842) following the manufacturer’s protocol. Anti-mouse Pannexin 1 antibody was purchased from CST (91137). The cleaved Pannexin 1 protein fragment was considered to be a marker of irreversible activation of the Pannexin 1 channel^51^.

### Statistical analysis

All statistical data were represented as the mean ± standard deviation (SD). Comparisons of two groups were performed by two-tailed Student’s *t*-tests and multiple groups were performed by one-way ANOVA with Tukey correction, *P* < 0.05 was considered statistically significant. All statistical analyses were performed by SPSS 23.0. All statistical graphs were drawn by GraphPad Prism 8.0.2.

## Supplementary information

Supplemental Information

Dynamic observation of C2C12 myoblasts fusion

Supplemental Video 1 Caption

## Data Availability

The data used and/or analyzed during the current study are contained within the manuscript or available from the corresponding author upon reasonable request.
