# Supplementary material for: MSCs-derived apoptotic extracellular vesicles promote muscle regeneration by inducing Pannexin 1 channel-dependent creatine release by myoblasts
Source: Int J Oral Sci. 2023 Jan 16;15:7. doi: 10.1038/s41368-022-00205-0 (PMC9842731; doi:10.1038/s41368-022-00205-0)
Supplement: Supplementary file 1 — Supplemental Information [file 41368_2022_205_MOESM1_ESM.docx]

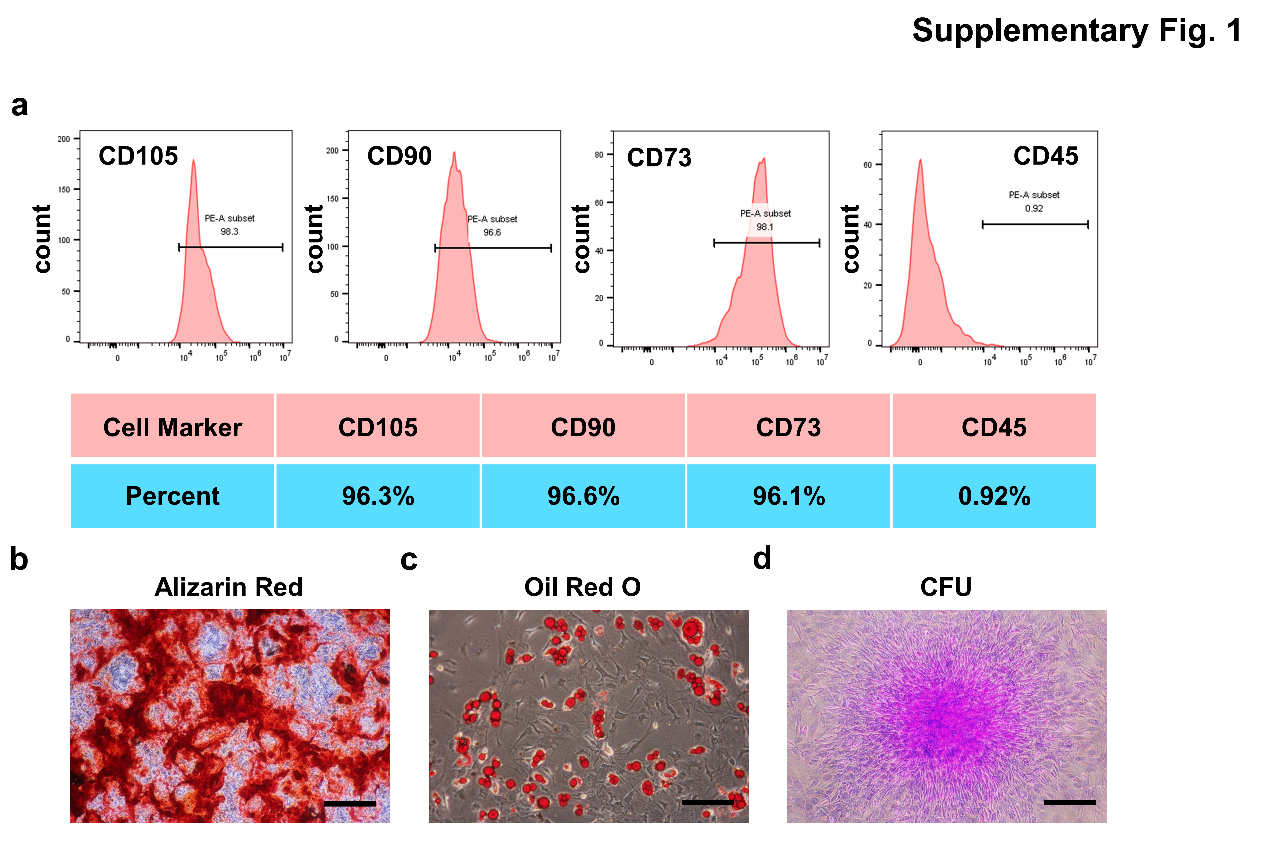


**Supplementary Fig. 1** Characterization of bone marrow derived MSCs **a-d.** Characterization of MSCs by flow cytometric analysis of cell markers such as CD105, CD90, CD73, and CD45 **(a)**. The representative images of alizarin red and oil red O staining to examine the multi-differentiation potential of bone marrow derived MSCs **(b, c)**. The representative image of colony formation of bone marrow derived MSCs **(d)**. The scale bar indicates 200 μm in image **b** and 100 μm in image **c** & **d**.


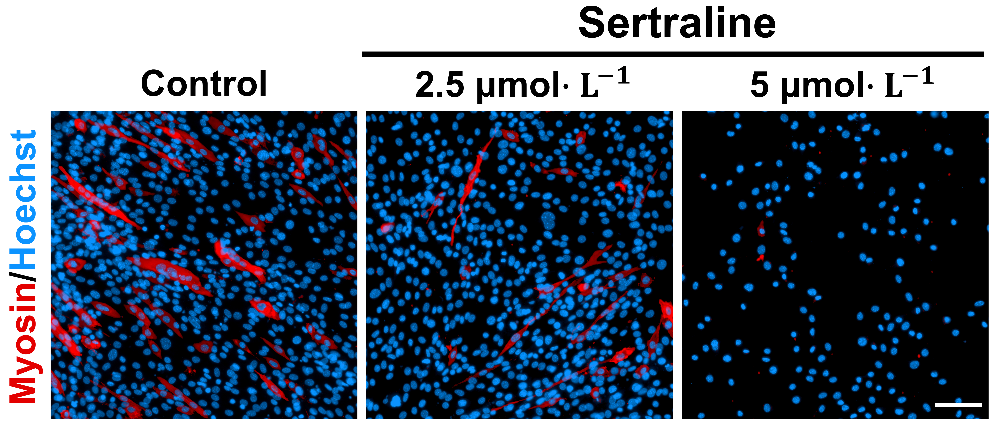


**Supplementary Fig. 2** Sertraline inhibits C2C12 myoblasts fusion *in vitro*. **a.** The representative immunofluorescence images of myosin staining of C2C12 myoblasts in control and the sertraline-treated groups. **b.** The analysis of the fusion index of C2C12 myoblasts in the different treated groups. n=3 per group; data were shown as mean ± SD; ns, not significant; the scale bar indicates 20 μm; **P* < 0.05; ***P* < 0.01; ****P* < 0.001.
