# Supplementary material for: MSCs-derived apoptotic extracellular vesicles promote muscle regeneration by inducing Pannexin 1 channel-dependent creatine release by myoblasts
Source: Int J Oral Sci. 2023 Jan 16;15:7. doi: 10.1038/s41368-022-00205-0 (PMC9842731; doi:10.1038/s41368-022-00205-0)
Supplement: Supplementary file 3 — Supplemental Video 1 Caption [file 41368_2022_205_MOESM3_ESM.docx]

**Video. 1** Dynamic observation of C2C12 myoblasts fusion. C2C12 myoblasts in fusion medium with TO-PRO-3 (indicating Myo-ApoEVs by detecting nucleic acid through activated Pannexin 1 channel) were dynamically observed by PerkinElmer Operetta CLS and the picture was taken every 15 min.
